# Studies in Zebrafish Demonstrate That *CNNM2* and *NT5C2* Are Most Likely the Causal Genes at the Blood Pressure-Associated Locus on Human Chromosome 10q24.32

**DOI:** 10.3389/fcvm.2020.00135

**Published:** 2020-09-02

**Authors:** Krishan K. Vishnolia, Celine Hoene, Karim Tarhbalouti, Julian Revenstorff, Zouhair Aherrahrou, Jeanette Erdmann

**Affiliations:** ^1^Institute for Cardiogenetics, University of Luebeck, Luebeck, Germany; ^2^DZHK (German Research Centre for Cardiovascular Research), Partner Site Hamburg/Luebeck/Kiel, Luebeck, Germany; ^3^University Heart Centre Luebeck, Luebeck, Germany

**Keywords:** blood-pressure, zebrafish, GWAS & Post-GWAS, 10q24.32 locus, *nt5c2* & *cnnm2*

## Abstract

**Background:** Globally, high blood pressure (BP) is the most important risk factor for cardiovascular disease. Several genome-wide association studies (GWAS) have identified variants associated with BP traits at more than 535 chromosomal loci with genome-wide significance. The post-GWAS challenge is to annotate the most likely causal gene(s) at each locus. Chromosome 10q24.32 is a locus associated with BP that encompasses five genes: *CYP17A1, BORCS7, AS3MT, CNNM2*, and *NT5C2* and warrants investigation to determine the specific gene or genes responsible for the phenotype.

**Aim:** To identify the most likely causal gene(s) associated with BP at the 10q24.32 locus using zebrafish as an animal model.

**Results:** We report significantly higher blood flow, increased arterial pulse, and elevated linear velocity in zebrafish larvae with *cnnm2* and *nt5c2* knocked down using gene-specific splice modification transcriptional morpholinos, compared with controls. No differences in blood-flow parameters were observed after *as3mt, borcs7*, or *cyp17a1* knockdown. There was no effect on vessel diameter in animals with any of the four genes knocked down. At the molecular level, expression of hypertension markers (*crp* and *ace*) was significantly increased in *cnnm2* and *nt5c2* knockdown larvae. Further, the results obtained by morpholino knockdown were validated using zebrafish knockout (KO) lines with *cnnm2* and *nt5c2* deficiency, again resulting in higher blood flow, increased arterial pulse, and elevated linear velocity. Analysis of *nt5c2a* KO larvae demonstrated that lack of this gene resulted in reduced expression of *cnnm2a*, with reciprocal downregulation of *nt5c2a* in *cnnm2a* KO larvae. Staining of whole-blood smears from *nt5c2* mutants revealed that KO of this gene might be associated with an acute lymphoblastic leukemia phenotype, consistent with literature reports. Additional experiments were designed based on previous literature on *cnnm2a* mutant zebrafish revealed impaired renal function, high levels of renin, and significantly increased expression of the *ren* gene, leading us to hypothesize that the observed elevated blood-flow parameters may be attributable to triggering of the renin–angiotensin–aldosterone signaling pathway.

**Conclusion:** Our zebrafish data establish *CNNM2* and *NT5C2* as the most likely causal genes at the 10q24.32 BP locus and indicate that they trigger separate downstream mechanistic pathways.

## Introduction

Blood pressure (BP) is reportedly the strongest heritable risk factor for stroke and coronary artery disease (CAD), leading to its prominent role in global morbidity and mortality (1). Pressure exerted by circulating blood on the blood vessels (mainly the large arteries of the systematic circulation) is termed BP. As per American College of Cardiology (ACC)/American Heart Association (AHA) Hypertension guidelines (2017), BP was classified in four stages, i.e., normal BP at <120/80 mm Hg, elevated at 120–129/ <80 mm Hg, stage 1 hypertension 130–139/80–89 mmHg, and stage 2 hypertension at ≥140/90 mmHg (2). High BP itself was responsible for 7.8 million deaths and loss of 148 million disability life years worldwide in 2015 alone (1). Factors contributing to BP level in an individual can be only determined after taking into consideration complex interactions between his/her life course exposures and genetic predispositions, as shown by several familial studies (3–5).

The development of genome-wide association studies (GWAS) has ensured identification of numerous genetic variants and their associations with diseases. Nevertheless, these variants provide limited knowledge above heritability projected in previous family based studies; that is, there is missing heritability, which remains unidentified (3). Expansion of GWAS in recent years has broadened our understanding by identifying associations of numerous novel genetic variants with specific diseases. Evangelos et al. recently conducted the largest GWAS for BP traits in over one million people of European ancestry and reported the identification of 535 novel loci, representing new biological insights into BP (6).

There is a plethora of literature available describing the overlap and association between cardiovascular diseases and BP (6–8). In a recent publication, Evangelos et al. reported associations of angiogenesis and vascular smooth muscle cell regulating genes, such as vascular endothelial growth factor A (*VEGFA*), fibroblast growth factor (*FGF5*), and *FGF9*, with BP (6). Lipid-related genes, such as apolipoprotein E (*APOE*), low-density lipoprotein receptor-related protein 4 (*LRP4*), and apolipoprotein L domain containing 1 gene (*APOLD1*), were also observed to have strong associations with multiple cardio-metabolic traits (6).

The zebrafish, *Danio rerio*, is a small tropical freshwater fish that lives in the rivers of northern India, northern Pakistan, Nepal, and Bhutan. The small size and ease of culture of this organism (9) mean that it is well-suited to developmental and genetic analyses, and it has become established as a powerful model organism for the study of vertebrate biology over the past 20 years. Additional advantages of zebrafish include their ability to produce large numbers of offspring (200–300, compared with an average for mice of 5–10 offspring), the fact that they are genetically similar to humans (90% at the genomic level) (10), and the ease with which their genes can be manipulated in the laboratory (11). Further, a crucial factor in the establishment of zebrafish as an animal model is that their larvae remain optically transparent during the first month of development, enabling temporal observation of developmental biology, fluorescent proteins, and probes in living animals (12).

Exploitation of the optical transparency of zebrafish larvae has led to the development of the platform MicroZebraLab software (ViewPoint), which allows non-invasive simultaneous measurement of differential cardiac chamber beat frequencies, blood-flow rates, vasodilation/constriction, stroke volume, and linear velocity (13). The rapid development of the two-chambered zebrafish heart includes the beginning of contractions, as early as 26 h post-fertilization (hpf), initiation of looping by 48 hpf, and full vascular tree development by 72 hpf (14). The adult zebrafish action potential presents similarities with that of humans; for example, the zebrafish action potential upstroke is led by Na^+^ channels and L-type Ca^2+^ channels, which are important for the plateau phase. Although zebrafish do not possess functional slowly activating K^+^ currents, the presence of cardiac T-type Ca^2+^ currents suggests that the electro-physiology of the adult zebrafish may be comparable to the human fetal phenotype (15). Vascular development begins with the migration of angioblasts at 24 hpf, which initiate the formation of two major axial vessels, the dorsal aorta, and the posterior cardinal vein, which are fully formed and begin to carry blood at 30 hpf (13). Even at 20 days post-fertilization, very few vascular smooth muscle cells can be detected in the caudal vein, probably because venous control of BP is less critical in fish than in terrestrial animals, where gravitational influences are greater (13).

Zebrafish serves as a simple vertebrate model, enabling profiling of novel cardiovascular drugs before initiating mammalian toxicity tests. Recent study investigated the translational power of zebrafish (in a meta-analysis) in comparison to rat, dog, and human to three model compounds (propranolol, losartan, and captopril), which act as modulators on two key systems (beta-adrenergic and renin–angiotensin systems) regulating cardiovascular functions (16). Results showed that the zebrafish cardiovascular responses were highly similar (over 80%) to those in humans against the model compounds, both in direction and in effect size. Considering these results and all the advantages mentioned earlier, zebrafish sets out to be an ideal model system for early-stage cardiovascular and/or blood-flow investigations (16).

**Figure 1:**
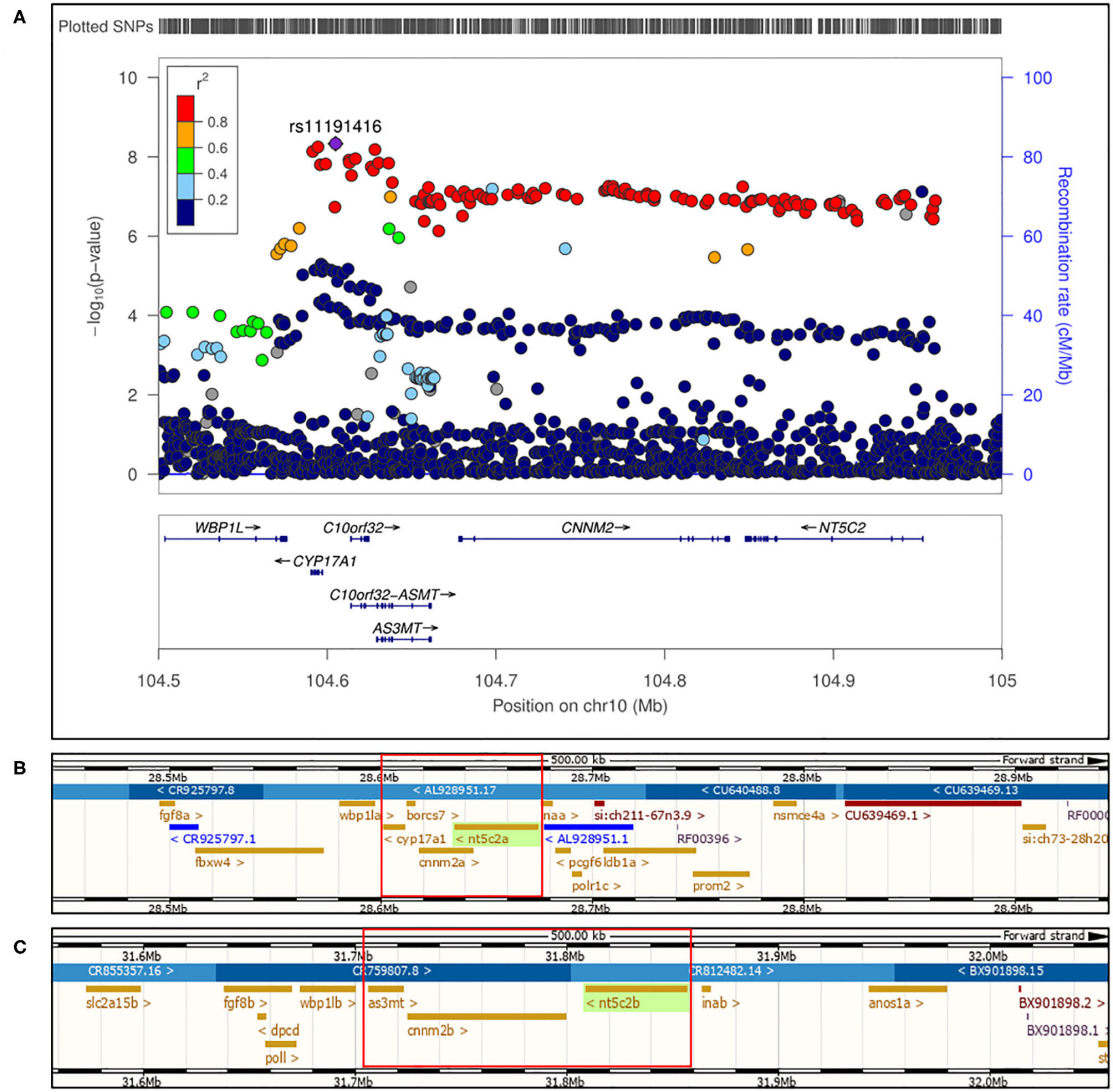
**(A)** Regional association plot for human locus 10q24.32 on chromosome 10 showing genome wide significant single nucleotide polymorphisms for blood pressure phenotype. Figure adapted from (17). **(B)** First part of 10q24.32 locus conserved in zebrafish genome at chromosome 13 encompassing “a” paralogs of *cnnm2, nt5c2*, and *cyp17a1* with *borcs7*. **(C)** Second part of 10q24.32 locus region conserved in zebrafish genome at chromosome 1 encompassing “b” paralogs of *cnnm2* and *nt5c2*, along with *as3mt* genes.

As mentioned above, hundreds of genes and loci associated with BP phenotypes have been identified by GWAS; however, limited functional studies to support the GWAS findings have been reported to date. Most loci associated with a BP phenotype encompasses a number of genes, while understanding of which gene (or genes) may be causal for the phenotype, and whether they are protective or detrimental, is lacking. Here, we conducted a proof-of-principal study, which could serve as a blueprint for the investigation of several other loci associated with BP, using zebrafish as a model organism. In the current study, we chose to dissect the chromosome 10q24.32 BP locus, which encompasses five genes, *CYP17A1, BORCS7 (labeled as C10orf32), AS3MT, CNNM2*, and *NT5C2*^17^ (Figure 1), with the aims of (1) determining the causal gene(s) specific for the BP phenotype and (2) unraveling the pathomechanisms linking the gene(s) to the phenotype.

Newton-Cheh et al. in 2009 identified 10q24.32 locus in human to be genome wide associated with blood pressure phenotype (17). Genes encompassed on locus 10q24.32 from human were found to be conserved in zebrafish on two different chromosomes along with *NT5C2* and *CNNM2* genes having two paralogs each. As highlighted in Figure 1, in zebrafish genome *cnnm2a, cyp17a1*, and *nt5c2a* genes were encompassed on chromosome 13 (Figure 1B) and *as3mt, cnnm2b*, and *nt5c2b* genes were encompassed on chromosome 1 (Figure 1C). As per ensemble database chromosome 13 in the zebrafish genome, the *borcs7* gene was based in between *cyp17a1* and *cnnm2a*; therefore, we included it too in our investigations. A publicly available eQTL database along with one eQTL search engine developed and published by our lab, i.e., Qtlizer from Genehopper (18), was used to determine the beta values associated with the risk alleles for each gene. All the five genes included in current study revealed both positive and negative beta values suggesting no concrete indication toward the expression of individual gene.

## Methods

### Zebrafish Maintenance

Animal studies were performed in accordance with the guidelines of the animal studies committee of Schleswig-Holstein, Germany. AB wild-type, *cnnm2a*^*sa*14149^, *cyp17a1*^*sa*22316^, and *nt5c2a*^*sa*17723^ strains were maintained under standard conditions at Fraunhofer Institute of Marine Biotechnology (EMB, Luebeck, Germany), as previously described in The Zebrafish book (19, 20). The zebrafish mutant lines, *cnnm2a* and *cyp17a1*, were purchased from the Zebrafish International Resource Center (ZIRC, University of Oregon, USA). AB wild-type and *nt5c2a* mutant lines were purchased from the European Zebrafish Resource Center (EZRC, Germany).

### Genotyping

The genotyping was performed according to previously described protocol (19), following which DNA was extracted using the DNeasy Blood and Tissue Kit (Qiagen, Germany), as per the manufacturer's protocol. Fragments containing mutations were amplified using specific genotyping primers. PCR conditions are provided in Supplementary File 1, and primer sequences are listed in Table 1. Following PCR, samples from *cyp17a1* mutants were sent for Sanger sequencing at Seqlab/Microsynth, Germany, whereas amplicons from *cnnm2a* and *nt5c2a* mutants were genotyped by restriction fragment length polymorphism (RFLP), which involved digestion with TaqI and BseMII (Thermo Fisher, Germany) enzymes, respectively, followed by electrophoresis of the digested products on 2% agarose gels alongside marker of molecular weight of 100 bp.

**Table 1:** Table below consists of all the primer (forward and reverse) names and their respective sequences used during the whole study.

**Gene name**	**Sequence**	**Purpose**
*cnnm2a_F*	TTGTGCAGGACACTCTTTGC	RFLP
*cnnm2a_R*	TGATGAAAACACAAGCCAACA	RFLP
*nt5c2a_F*	TCTTTAACAGTTGCAAACCATGA	RFLP
*nt5c2a_R*	CCTTGAGGATTGGAGAAAACA	RFLP
*cyp17a1_F*	GGAATGGAAGAACCCTGAGC	Sequencing
*cyp17a1_R*	ACTGAGCGCCGTCTGAAAT	Sequencing
*nt5c2a_F*	CAACTTCTTGCGTGGTCCAG	qPCR for MO-KD efficiency
*nt5c2a_R*	GGACCCAATCCACAGCATCT	qPCR for MO-KD efficiency
*cyp17a1_F*	TCAGCGACAGGGGGAATCTA	qPCR for MO-KD efficiency
*cyp17a1_R*	TTCAGAAAGCGTCCTGGGTC	qPCR for MO-KD efficiency
*cnnm2a_F*	GTCAGCAGGGCAGAATCACT	qPCR for MO-KD efficiency
*cnnm2a_R*	GGCGGGTCACCTTGATGTAG	qPCR for MO-KD efficiency
*borcs7_F*	CTACTCAGTCAGGCAGCGAG	qPCR for MO-KD efficiency
*borcs7_R*	GGTACTGCAAGTGGGTCGTT	qPCR for MO-KD efficiency
*as3mt_F*	GGCACGTCACAGGTATCGAC	qPCR for MO-KD efficiency
*as3mt_R*	CCTCCGTCCTTCAGAACACAA	qPCR for MO-KD efficiency
*nt5c2b_F*	AGCAGTGTACAAATCGCCTGA	qPCR for MO-KD efficiency
*nt5c2b_R*	CAGGCCCTCGCAGAAAGTTA	qPCR for MO-KD efficiency
*cnnm2b_F*	TTCAGTCCCAGCCAGATGTC	qPCR for MO-KD efficiency
*cnnm2b_R*	AGCCCTACTGACCACAAAGG	qPCR for MO-KD efficiency
*nt5c2a_F*	GGTGCATCACAGAGTTTTTGTCA	RT
*nt5c2a_R*	CACCAAGCCTCTTGTGGGAA	RT
*cyp17a1_F*	GAGGCCACGGACTGTTACAA	RT
*cyp17a1_R*	CACACATAGAGCTCGCCTCC	RT
*ren_F*	GGGGCTTTCTGAGTGAGGAC	RT
*ren_R*	ATATCTGCCCCAACCGACAC	RT
*ace_F*	GCTGGGCACTGACAAAATGG	RT
*ace_R*	TCGGTGTAAGCGTTCCAGAC	RT
*crp_F*	TGCTTCAGTTCAAGACGGCTA	RT
*crp_R*	AACGCTGCATCCTTACTAGACTG	RT
*EF1α_F*	CTGGAGGCCAGCTCAAACAT	RT
*EF1α_R*	ATCAAGAAGAGTAGTACCGCTAGCATTAC	RT
*β-actin_F*	CGAGCTGTCTTCCCATCCA	RT
*β-actin_R*	TCACCAACGTAGCTGTCTTTCTG	RT

### Morpholino Injections

Transcriptional splice modification morpholinos (5 ng/embryo) were injected into freshly hatched one-cell-stage AB wild-type zebrafish embryos. The following morpholinos were custom designed and purchased from Gene Tools (USA): *as3mt* (5′-AGTCCTGTCCCTTTGAACAAGAAAT-3′), *borcs7* (5′-ATGTGTCGTATATTTACTTCTTGGC-3′), *cnnm2a* (5′-TCAGTGGATTAATTTCTGACCTGCA-3′), *cnnm2b* (5′-CAAGACGTTCATGGCACATACTATA-3′), *cyp17a1* (5′-TGCAGTGACACTCACTTATCTTCTC-3′), *nt5c2a* (5′-AGATACTTGTTGATCTTACCTGCCA-3′), and *nt5c2b* (5′-GTCCATTTATTTGTCTGATTTACCT-3′). Quantification of genetic downregulation was performed by quantitative real-time PCR (real-time qPCR) using custom-designed primers (Table 1).

The efficiency of morpholino knockdown in larvae was assessed individually after measurement of blood-flow parameters, using real-time qPCR at the mRNA level. In this study, three biological samples were analyzed in triplicate for all real-time qPCR experiments. Total RNA was extracted using RNeasy kit (Qiagen, Germany), following the manufacturer's protocols. *EF1*α and β*-actin* were used as internal housekeeping genes. Results were analyzed using the comparative threshold cycle method (2^−ΔΔCt^), to compare expression levels in samples and controls, where control samples were set to a value of 1.

### Blood-Flow Analysis

**Figure 2:**
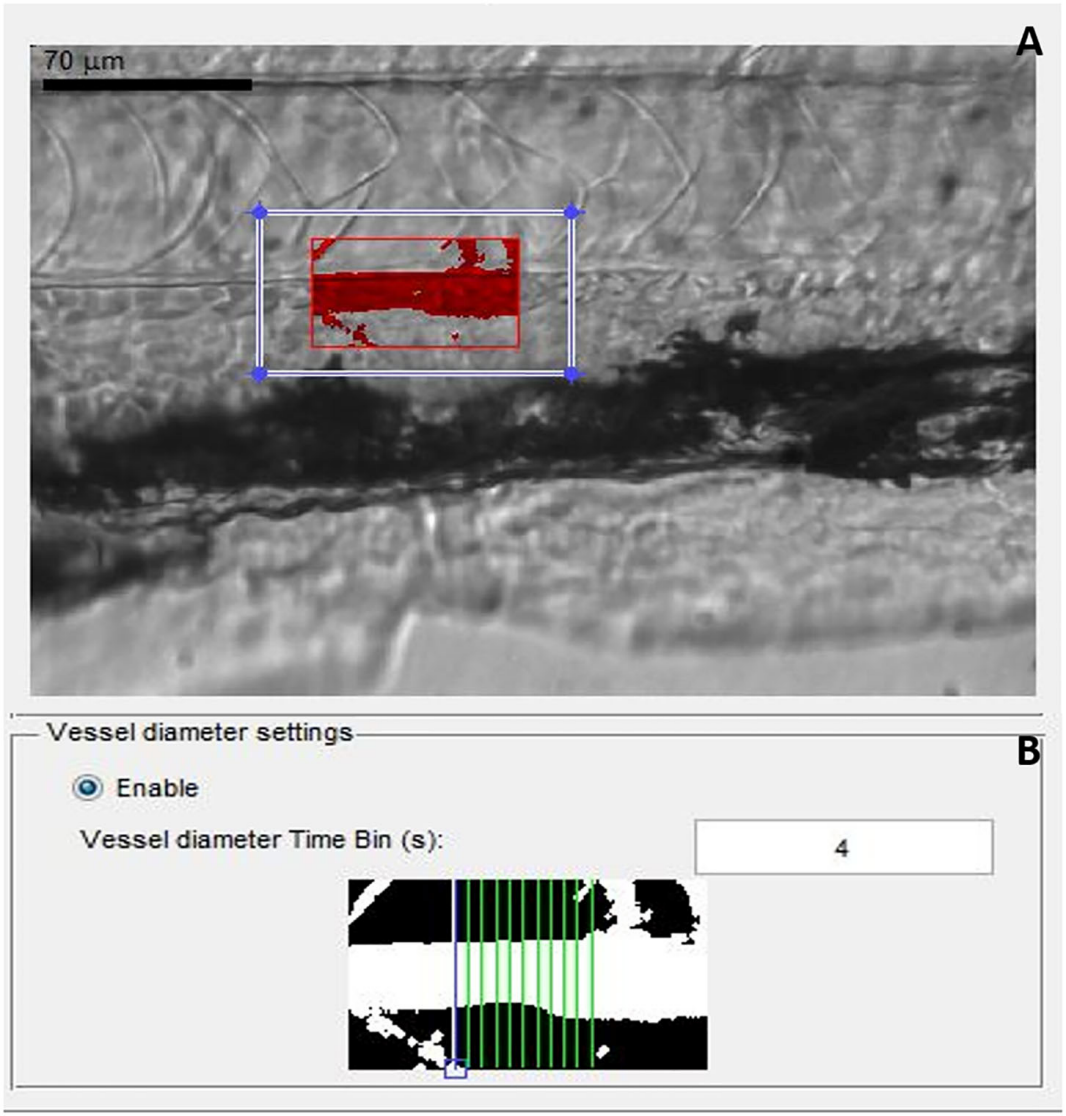
Screen shot images of MicroZebraLab software while recording a video file for analyzing blood flow parameters from zebrafish. **(A)** Red box represents the area of dorsal aorta selected manually for preparing the video, **(B)** gray scale image from the software showing measurement of vessel diameter from where the blood flow parameters are measured.

The protocol for blood-flow measurement and analysis was adapted from (13). At 72 hpf, individual larvae were transferred into 80 μl (total volume) of 0.1% low melting point agarose gel prepared in embryo system water, previously maintained at 17°C in a 1.5-ml Eppendorf tube. Larvae in low melting point agarose gel were then transferred onto coverslips, followed by transfer onto an inverted microscope (Zeiss stereo, Discovery V20, Germany), mounted with high-speed video cameras (GRAS-03K2M-C, Point Gray, Richmond, Canada). Larvae were positioned, and a region of interest was selected, focused to capture the dorsal aorta, caudal to the swim bladder, and on top of the anal orifice, at 120 frames *per second* (fps) for 20 min. The region of interest captured by the camera for video preparation is shown in Figure 2.

Once all the blood-flow videos were prepared for each group, in parallel with controls, they were analyzed using MicroZebraLab (Version 3.6, ViewPoint, Lyon, France), which detects changes in pixel density and combines them with vessel diameter to generate a flow rate (nl/sec) for every frame. Prior to analysis, the software was calibrated using a video recording of a hemocytometer grid, to establish the true width of the field of vision in microns. An area of each aortic blood vessel was carefully selected, avoiding any nearby capillaries, which might interfere with assessment of blood flow in the main vessel. Movement of erythrocytes within the tracked areas was detected by the software and used to compute the blood flow. In parallel, vessel diameter measurements were conducted by the software every 20 s, following selection of a section of vessel and confirmation of two vessel edges. During analysis of video files to determine blood-flow parameters, the first 3 min of every video file was deleted, as this is the time needed for the zebrafish larvae to acclimatize to the conditions, as demonstrated in a previous publication (13) and also confirmed in our hands (data not included here). Blood-flow parameters were blindly measured from individual larvae for all the experiments without knowing their genotype, following which larvae was snap frozen for genotyping. After genotyping of individual larvae, data was statistically analyzed and plotted in graphs from heterozygote, homozygote, MO-knocked down larvae, and wild types.

### Ramipril Treatment

Ramipril is an angiotensin-converting enzyme inhibitor used as a first-line medication for patients with high BP or congestive heart failure (21, 22). Zebrafish larvae (72 hpf) were first treated with ramipril in five groups (*n* = 25 larvae per group) at 0.1, 0.2, 0.3, 0.4, and 0.5 nM, to determine the most effective concentration, to be used in all subsequent experiments. To investigate its effects (i.e., whether blood-flow phenotype could be rescued), AB wild-type, *cnnm2a*, and *nt5c2a* mutant larvae were treated with ramipril for an hour before measurement of blood-flow parameters.

### Adult Zebrafish Blood Smear Staining

Adult *nt5c2a, cnnm2a* mutants and AB wild-type zebrafish (*n* = 6 per group) were anesthetized and sacrificed with the lethal dose of tricaine solution (0.1 mg/ml). Small blood samples (i.e., ≤ 0.4% of body weight) were collected from individual zebrafish, following the published protocol (23). Collected blood samples were smeared on normal glass slides (Thermo Fisher Scientific, Germany) and left to dry overnight. Following the manufacturer's protocol for May–Grünwald–Giemsa staining, slides were incubated in May–Grünwald stain (Merck, Germany) for 4 min followed by incubation in a 1:1 solution of May–Grünwald stain and May–Grünwald buffer. After washing with May–Grünwald buffer, slides were incubated in Giemsa stain for 20 min, followed by washing with Giemsa buffer. Slides were air dried for a few hours before imaging under an inverted microscope (BZ9000, Keyence, Germany).

### Renin ELISA

ELISAs were performed using the Mouse REN (Renin) ELISA kit (E-EL-M0061.96, Elabscience, Germany), according to the manufacturer's protocol. Most of the antibodies and kits used in zebrafish research are from mice or other species because of the species cross-reactivity; similarly, Mouse REN ELISA Kit was mentioned by the manufacturers to be working with zebrafish samples. Homozygote *nt5c2a KO* (*n* = 10), *cnnm2a KO* (*n* = 10), and AB wild-type (*n* = 10) larvae (96 hpf) were homogenized in 300 μl of PBS in six groups per genotype (*n* = 60 per genotype), to investigate renin levels. All analyses and calculations were conducted following the protocol provided by the ELISA kit manufacturer.

Renin levels were also investigated by extracting RNA from 96 hpf larvae (*n* = 6 larvae per group) and performing real-time qPCR for *ren* gene expression.

### Renal Function Assay

We also investigated the kidney function of *cnnm2a* mutant zebrafish using a fluorescent clearance assay, where the optical transparency of the zebrafish was exploited to quantitatively monitor the clearance of fluorescent dye from the vasculature and out through the kidney over time, to provide a measure of kidney function (24). Using a microcapillary, 1 nl of Rhodamine B isothiocyanate-dextran (RITC-Dextran) (10,000 MW) dye (5 mg/ml) was microinjected into the pericardial cavity of 96 hpf larvae (*n* = 20 per group). Fluorescence and bright-field images were captured in parallel from all groups (heterozygote, homozygote, and wild-type) after 3, 12, and 24 h following the injections. All images were captured using the inverted Keyence microscope at 40 times magnification.

### Statistical Analysis

All experiments were statistically analyzed using the program GraphPad Prism 6. After testing the normality, data (represented as mean ± SD) from experiments with only two groups were analyzed by non-parametric Student's *t*-test, while data from experiments with three or more groups were compared using two-way ANOVA, with multiple comparisons and without correction. The significance alpha *p*-value was adjusted by dividing it with the total number of tests performed.

## Results

All five genes encompassed in the human chromosome 10q24.32 and CAD and BP locus were found to be highly conserved between zebrafish and humans at both the cDNA and amino acid levels, respectively: *as3mt*, 60.98% and 73%; *borcs7*, 75.53% and 65.84%; *cnnm2a*, 76.66% and 84%; *cyp17a1*, 47% and 65%; and *nt5c2a*, 77.47% and 95%. Further, the “*b*” paralogs of the *cnnm2* and *nt5c2* genes were more than 90% identical to the “*a*” paralog according to the Ensembl database.

### Efficiency of Knockdown Using Morpholinos

**Figure 3:**
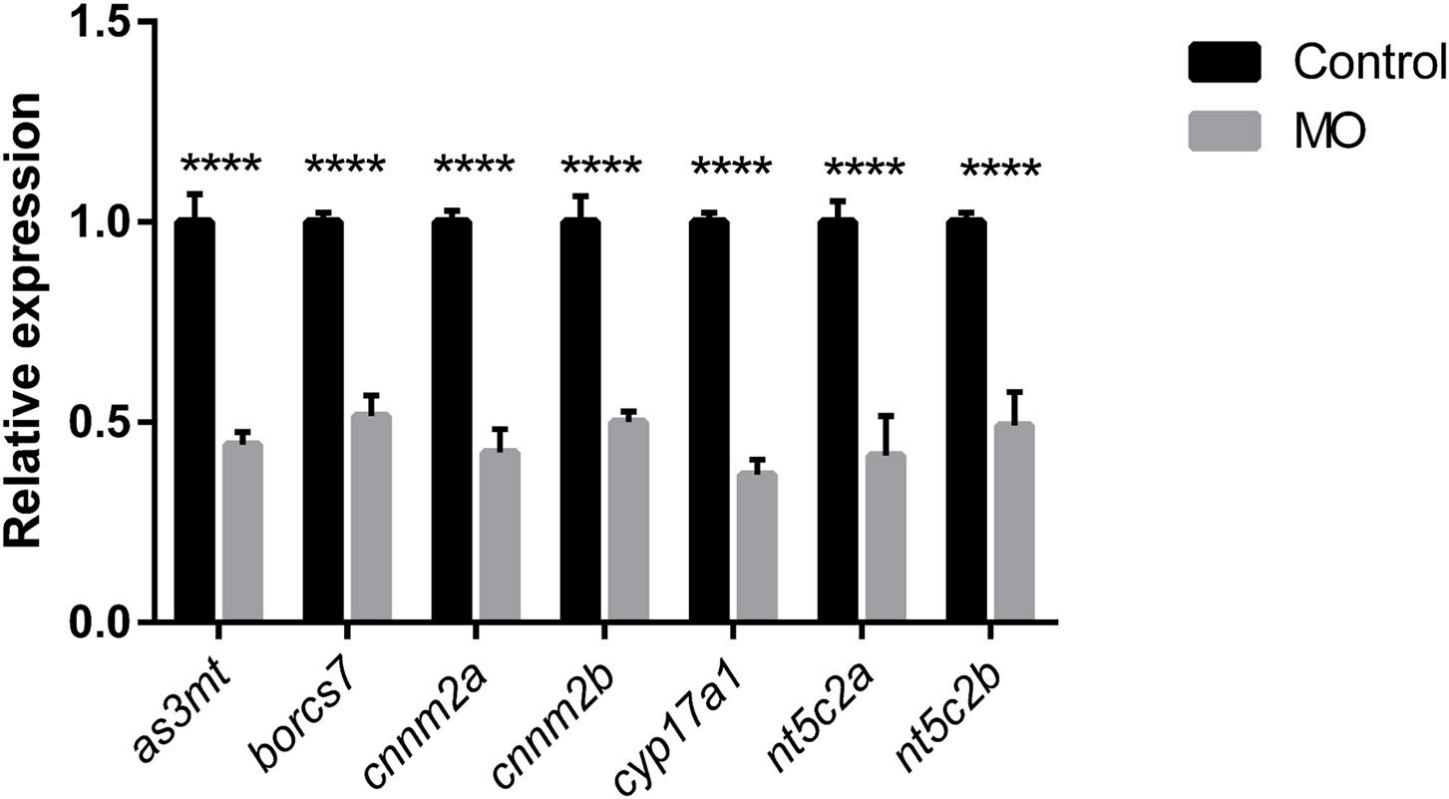
Graph represents downregulation of the relative gene expression of *as3mt, borcs7, cnnm2a, cnnm2b, cyp17a1, nt5c2a*, and *nt5c2b* measured at 72 h post morpholino injections compare to their respective controls injected with control morpholinos from *n* = 10 larvae per group. Error bars represents standard deviation and significance was set at *p* < 0.05. **** represent the significant differences among the samples.

Quantification of genetic knockdown in larvae injected with splice modification transcription morpholinos was investigated at 72 hpf by measuring relative gene-expression levels compared with controls using real-time qPCR. We identified similar genetic downregulation, in the range of ~40–50%, using all our morpholinos (Figure 3).

### Determination of Blood-Flow Parameters in Larvae Following Morpholino Knockdown

**Figure 4:**
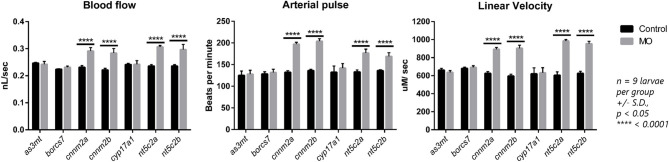
Graphs represents blood flow parameters measured after downregulation of *as3mt, borcs7, cnnm2a, cnnm2b, cyp17a1, nt5c2a*, and *nt5c2b* genes in zebrafish using transcriptional splice modification morpholinos. Blood flow (nL/sec), arterial pulse (beats per minute) and linear velocity (μM/sec) compared against their respective control groups. Data was generated from *n* = 9 larvae per groups, error bars represents standard deviation and significance was set at *p* < 0.05.

We observed significant increases in blood-flow parameters (i.e., blood flow, arterial pulse, and linear velocity) after genetic knockdown of the *cnnm2* and *nt5c2* genes for both “a” and “b” paralogs using morpholinos. In contrast, no differences were detected on knockdown of the *as3mt, borcs7*, and *cyp17a1* genes. Further, no differences were observed in vessel diameter following knockdown of any of the genes (Figure 4).

RNA was extracted for gene-expression analysis from the same individual larvae used for measuring blood-flow parameters. Expression of molecular markers for hypertension and inflammation (25), including angiotensin-converting enzyme (*ace*) and C-reactive protein (*crp*), was significantly increased following knockdown of both *cnnm2* and *nt5c2* (Table 2).

**Table 2:** Gene expression of hypertension and inflammation markers, i.e., *ace* and *crp*, post morpholino knockdown of *cnnm2a, cnnm2b, nt5c2a*, and *nt5c2b* genes in zebrafish.

**Gene knocked down**	**Gene of interest**	**Relative expression**	
*cnnm2a*	*ace*	Control: MO:	1 ± 0.169 1.498 ± 0.163*
	*crp*	Control: MO:	1 ± 0.124 2.15 ± 0.186*
*cnnm2b*	*ace*	Control: MO:	1 ± 0.124 1.375 ± 0.143*
	*crp*	Control: MO:	1 ± 0.112 1.98 ± 0.118*
*nt5c2a*	*ace*	Control: MO:	1 ± 0.173 2.55 ± 0.4606*
	*crp*	Control: MO:	1 ± 0.102 2.31 ± 0.248*
*nt5c2b*	*ace*	Control: MO:	1 ± 0.132 2.14 ± 0.143*
	*crp*	Control: MO:	1 ± 0.241 1.97 ± 0.118*

*Data was generated and pooled from n = 6 individual larvae per group. Star denotes the significant differences among the samples compare to their respective controls along with ± standard deviation*.

### Validation of Blood-Flow Analysis in Zebrafish Knockouts

**Figure 5:**
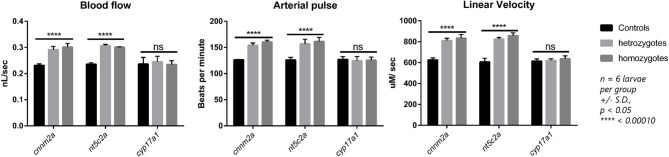
Blood flow parameters measured from heterozygote and homozygote *cnnm2a, nt5c2a*, and *cyp17a1* zebrafish mutants and compared against ab-wildtypes as control. Blood flow (nL/sec), arterial pulse (beats per minute) and linear velocity (μM/sec) were measured from *n* = 6 larvae per group and presented here as mean ± standard deviation and significance was set at *p* < 0.05.

Zebrafish mutant lines available for the *cnnm2a, cyp17a1*, and *nt5c2a* genes were purchased from international and European zebrafish resource centers. Validation experiments to evaluate blood-flow parameters in homozygote mutants of all three lines generated similar results, where *nt5c2a* and *cnnm2a* mutants exhibited significantly higher blood flow, arterial pulse, and linear velocity. No significant differences were observed in the blood-flow parameters of *cyp17a1* mutants, and there was no significant alteration in vessel diameter in mutants of any of the three genes (Figure 5).

### Phenotype Rescue by Treatment With Ramipril

**Figure 6:**
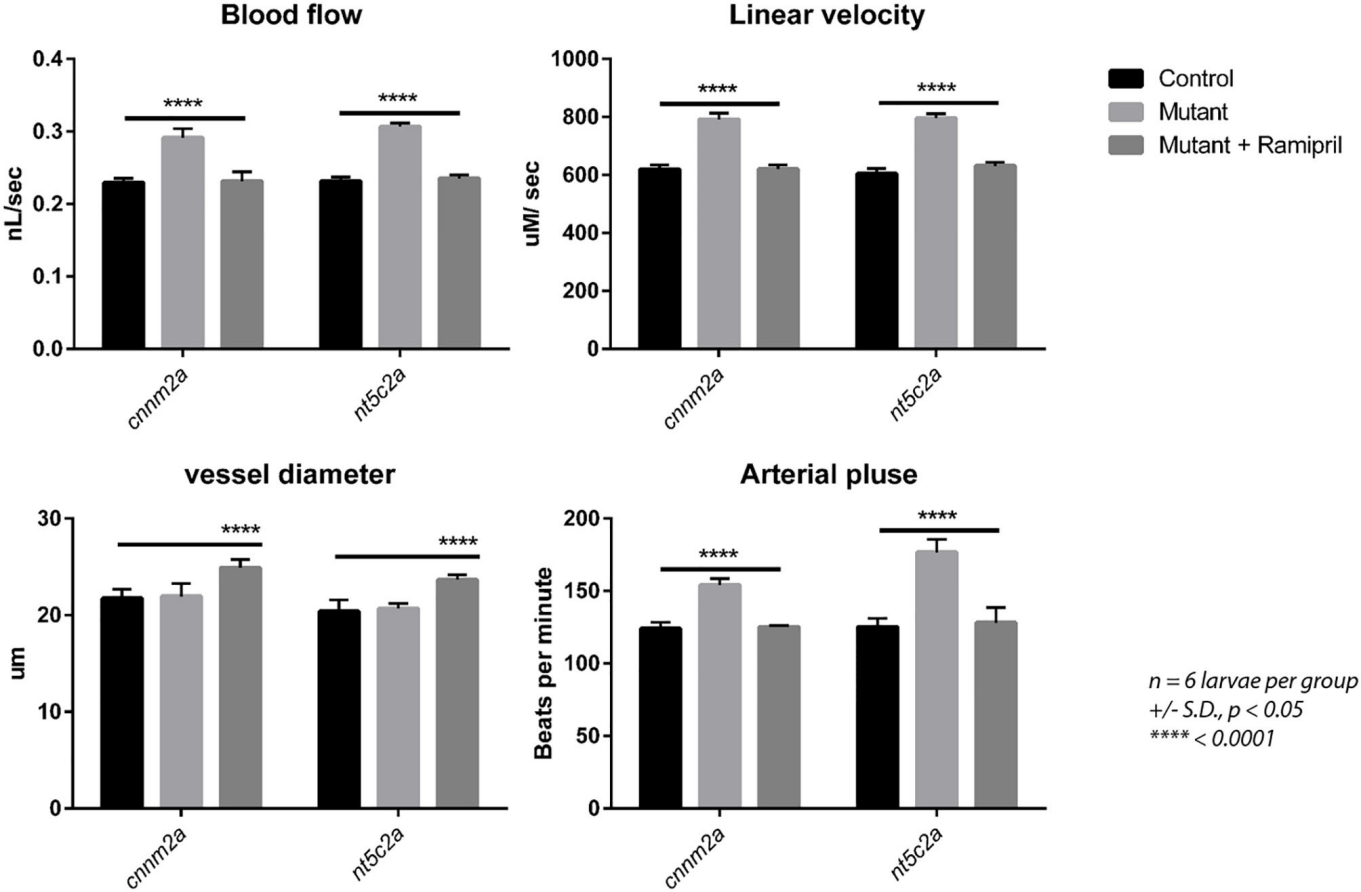
Blood flow parameters measured with and without Ramipril treatments from *cnnm2a* and *nt5c2a* mutant zebrafish larvae along with ab-wildtypes as control. Blood flow (nL/sec), arterial pulse (beats per minute), vessel diameter (μm) and linear velocity (μM/sec) were measured from *n* = 6 larvae per group and presented here as mean with standard deviation as error bars and significance was set at *p* < 0.05.

Ramipril is a well-known vasodilator used as a first-line medication for hypertensive patients (21). 0.3 nM concentration of ramipril was determined to be the most effective concentration for zebrafish larvae (Ab wild type), as the larvae treated with 0.4 and 0.5 nM ramipril did not survive (Supplementary File 2) and a non-significant effect on blood flow was observed from 0.1 and 0.2 nM-treated zebrafish larvae (Ab wild type). Therefore, 0.3 nM concentration of ramipril was used for all the subsequent phenotype rescue experiments (Figure 6). Zebrafish *cnnm2a* and *nt5c2a* mutants and control AB wild-type fish were treated with ramipril (0.3 nM) to investigate whether the blood-flow phenotypes of the mutants could be rescued by this drug. Blood-flow parameters measured from individual larvae (*n* = 6 per group) demonstrated significant changes in mutant larvae compared with controls. Treatment with ramipril attenuated the blood-flow parameters in both mutant lines, which did not differ significantly from those of control larvae following treatment. Vessel diameter was significantly increased in both *cnnm2a* and *nt5c2a* mutant larvae after treatment with Ramipril (Figure 6).

### Co-regulation of the nt5c2a and cnnm2a Genes in Zebrafish

**Figure 7:**
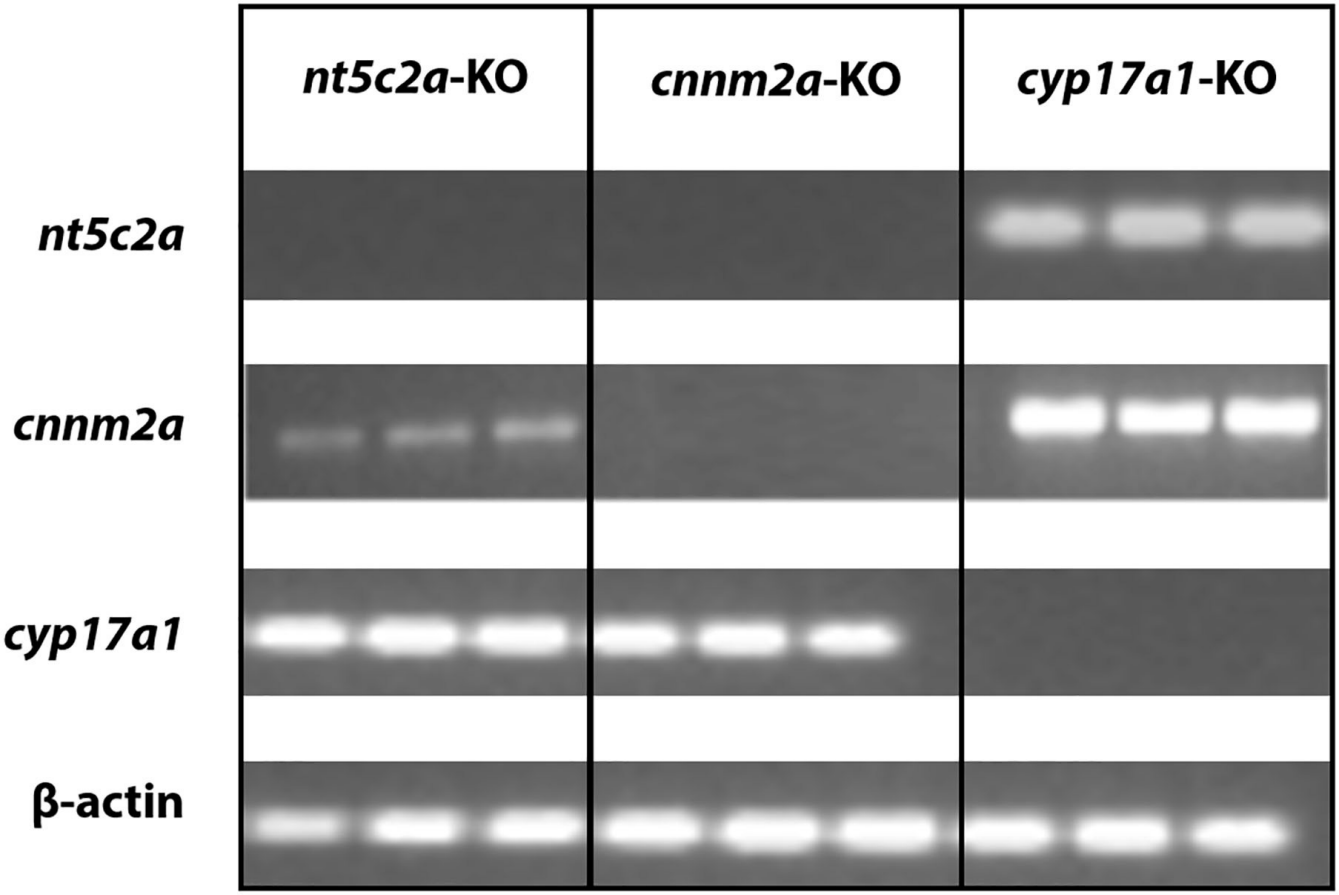
Co-regulation of cnnm2 and nt5c2 genes. Agarose gel micrograph representing bands of DNA samples collected from cnnm2a, cyp17a1, and nt5c2a mutants. Each set of samples were investigated for expression of other genes along with house-keeping genes.

Data mining using the 4C database browser indicated that there may be an interaction between the regulation of the *CNNM2* and *NT5C2* genes in humans (26). To investigate this possibility, we evaluated the expression of the *cnnm2a, cyp17a1*, and *nt5c2a* genes individually in zebrafish mutant larvae. We observed no expression of *nt5c2a* in the *cnnm2a* mutants and a significantly lower expression of *cnnm2a* in *nt5c2a* mutants (Figure 7). Both genes were expressed at normal levels in *cyp17a1* mutants, and the housekeeping gene β*-actin* was expressed at normal levels in all the mutants.

### Staining of Whole Blood Samples From nt5c2a Mutants

**Figure 8:**
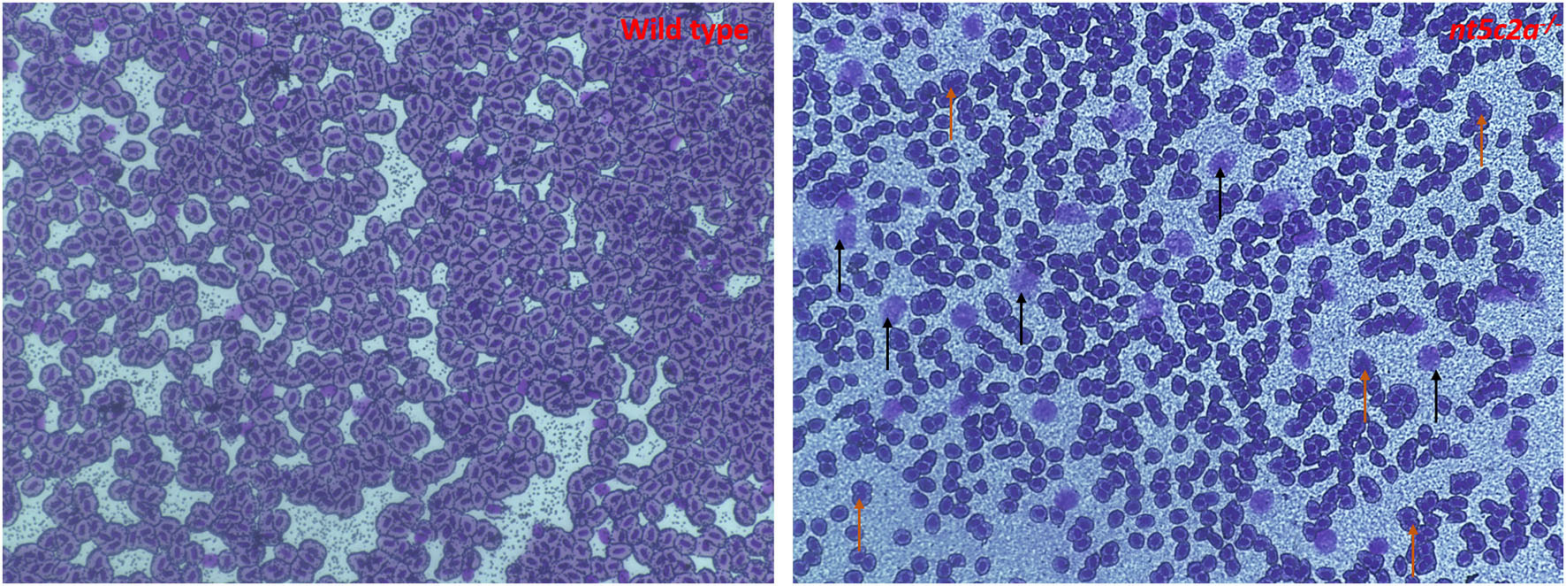
Microscopic images of May-Grünwald-Giemsa–stained blood smear from ab-wildtype and *nt5c2a* mutant (heterozygote and homozygote) zebrafish larvae (*n* = 6 per group). Black arrows on the stained image from *nt5c2a* mutant represents the groups of immature blood cells without any cytoplasmic material. Images were captured at 40X magnification. Red and black arrows on the stained image from nt5c2a mutants represents the group of multi-nucleated and immature blood cells without any cytoplasmic material respectively.

Blood was collected from adult *nt5c2a* homozygote mutant zebrafish and wild-type animals as controls (*n* = 6 per group). Blood samples were smeared on slides for May–Grünwald–Giemsa staining. As shown in representative images (Figure 8), numbers of immature or multinucleated blood cells (highlighted using brown and black arrows in Figure 8) were significantly higher in blood smears from *nt5c2a* mutant zebrafish relative to controls. This increased number of immature blood cells, lacking or disrupted cytoplasm around their nuclei, suggests an acute lymphoblastic leukemia (ALL) kind of phenotype, which has previously been reported with *nt5c2a* gene mutation (27, 28).

### Measurement of Renin Levels in cnnm2a Knockouts by ELISA

Renin levels were quantified in *cnnm2a* zebrafish larvae mutants and wild-type controls using a mouse ELISA, as species cross-reactivity meant that it could also be used for analysis of zebrafish (see Methods section). As shown in Figure 8, renin levels in *cnnm2a* mutant larvae were significantly higher than those in *nt5c2a* mutants and controls. Renin is a key player in the renin–angiotensin–aldosterone (RAAS) signaling pathway, which is associated with high BP (29, 30); therefore, the high levels of renin in *cnnm2a* mutant zebrafish larvae indicate a potential mechanism underlying the observed high blood flow in this model.

**Figure 9:**
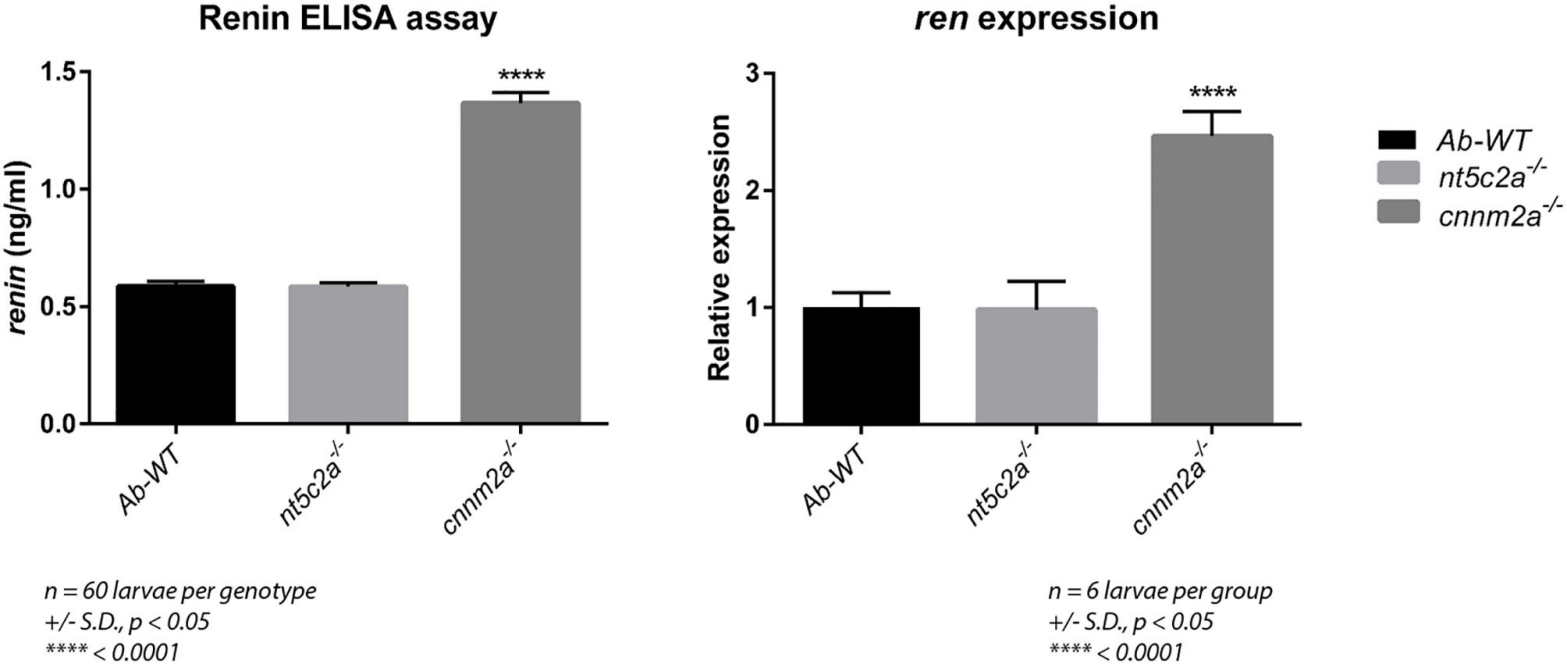
Graph on the left presents the renin levels (ng/ml) measured using ELISA assay from ab-wildtypes, *nt5c2a* and *cnnm2a* zebrafish mutant larvae. Assay was performed with six groups per genotype with *n* = 10 larvae per group (*n* = 60 larvae per genotype). Graph on the right presents relative expression of *ren* gene in ab-wild type, *nt5c2a* and *cnnm2a* mutant zebrafish larvae (*n* = 6 larvae per group). Data is presented as mean of the biological replicates with standard deviation in error bars. Significance was set at *p* < 0.05.

Increased renin levels in the *cnnm2a* mutants were also supported by a significant difference in the expression levels of the *ren* gene in the mutants relative to controls (Figure 9).

### Assessment of Kidney Function in cnnm2a Knockouts

**Figure 10:**
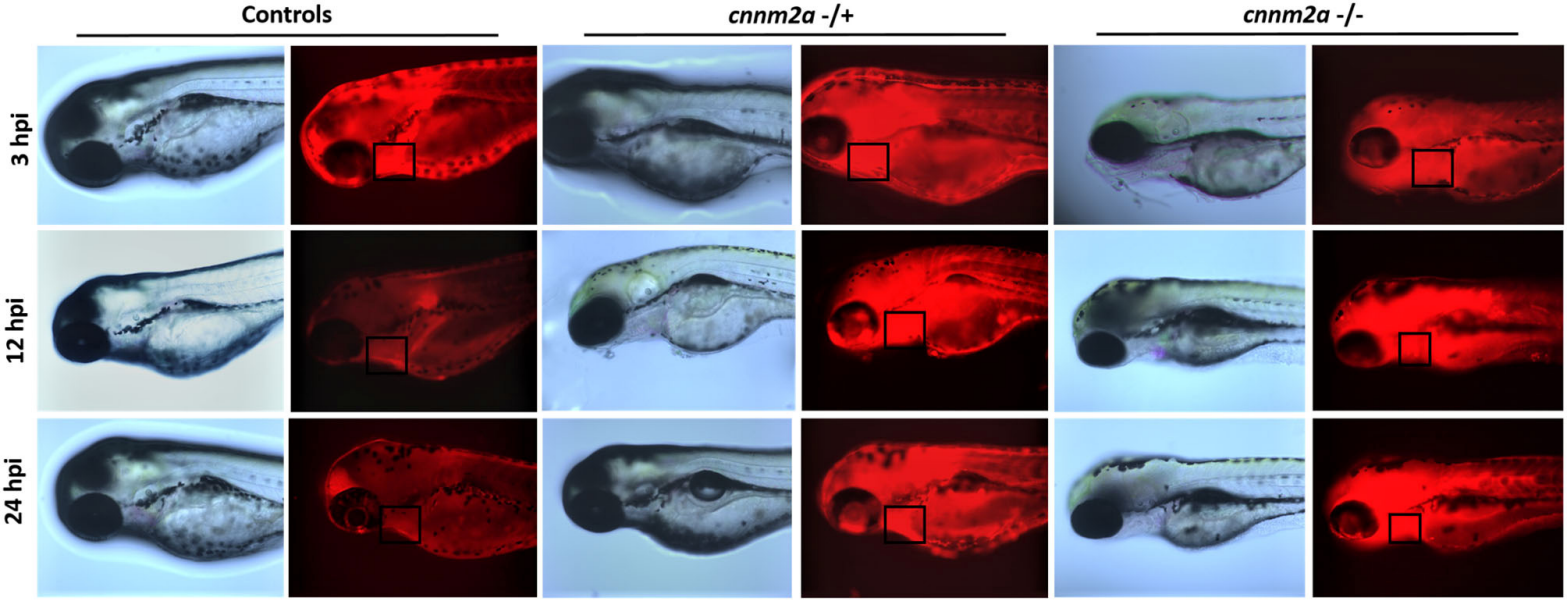
Zebrafish cnnm2a mutant (heterozygote and homozygote) larvae along with wildtype controls micro injected with rhodamine dextran dye in their pericardial sac. Bright field and fluorescent images captured at 3, 12, and 24 h post injection represent the inefficiency of cnnm2a mutant larvae to filter the dextran dye through kidney as the controls. Images were captured using Keyence inverted microscope at 40X magnification.

Fluorescent images collected after injection of RITC-Dextran dye into *cnnm2a* mutants and control larvae were compared at various time points: 3, 12, and 24 h postinjection. Images acquired at 3 h postinjection confirmed that the injections had penetrated the pericardial cavities of the zebrafish larvae. Under normal conditions, more than half of the initial fluorescence was reduced from the blood over a 24 h period by secretion via the kidney (24). The images in Figure 10 clearly show that *cnnm2a* mutants were unable to filter the injected dye through the kidney and clear it by 24 h postinjection, unlike control larvae. Inability to clear the dye in the normal manner suggests possible kidney dysfunction in the *cnnm2a* zebrafish mutants.

## Discussion

GWAS is a powerful tool for identification of novel genes and loci associated with a chosen phenotype. Evangelou et al. recently identified 535 novel loci associated with BP traits by analyzing over one million people (6). Moreover, a recent publication from our group reported 163 (and increasing) novel loci associated with CAD at a genome-wide significance level (31). A significant number of genes or loci are associated with both BP and CAD phenotypes (17). In the current study, we chose the 10q24.32 locus, which is genome-wide associated with BP and CAD. We decided to dissect the 10q24.32 locus, which encompasses five genes *CYP17A1, BORCS7, AS3MT, CNNM2*, and *NT5C2*, focusing on the BP phenotype, with the aim of understanding the underlying pathomechanisms.

We used zebrafish as a model organism, and all five genes from the human 10q24.32 locus were found to be conserved in the zebrafish genome on chromosomes 13 and chromosome 1 as represented in Figure 1. Genome duplication in zebrafish has led to duplicate paralogs of single copy genes (10). Accordingly, the *nt5c2* and *cnnm2* genes have two paralogs, “a” and “b,” in the zebrafish genome, mapping on completely different chromosomes (“a” paralog on chromosomes 13 and “b” paralog on chromosome 1, respectively) (10). We also observed *borcs7* gene to be located in between the *cyp17a1* and *cnnm2a* gene in chromosome 13 as highlighted in Figure 1B. Our initial experiments, using custom-designed transcription splice modification morpholinos targeting individual genes, generated interesting results, demonstrating that downregulation of *nt5c2* and *cnnm2* in zebrafish leads to significant alterations of blood-flow parameters; however, downregulation of the *as3mt, borcs7*, and *cyp17a1* genes did not result in any significant differences in those parameters. Further, no differences in vessel diameter were observed in response to downregulation of any of the five genes.

Transient downregulation using morpholinos provided useful data; however, further investigation to understand the underlying mechanism behind the observed differences in blood-flow parameters was warranted. Therefore, we purchased zebrafish mutant lines available from European or international zebrafish resource centers, including *cnnm2a, cyp17a1*, and *nt5c2a* gene knockout lines. Experiments to validate the results of the morpholino assays using the zebrafish mutant lines achieved similar results. Investigations from both *cnnm2a* and *nt5c2a* mutant zebrafish lead to the high blood-flow parameters; however, no difference in the vessel diameter lead us to postulate possible alterations in cardiac or renal mechanism.

We also investigated whether the high blood-flow phenotype of the zebrafish mutants could be rescued by treatment with the drug ramipril, a well-known vasodilator used as a first-line medication for humans with hypertension. Indeed, we observed dilation of blood vessels along with reversal of blood-flow parameters in the mutant larvae compared to wild types. However, it needs to be highlighted that ramipril rescues the blood-flow phenotype here via a completely different mechanism which is the direct vasodilatory effect.

Interestingly, there is a substantial literature supporting the potential of *CYP17A1* as a causal gene for hypertension. Association of *CYP17A1* with hypertension was first described in humans by Biglieri et al. in 1966, where they reported a gonad defect resulting in production of excess corticosterone and deoxycorticosterone and leading to hypertension (32). They also found an absence of aldosterone synthesis in subjects with normal stature and amenorrhea; however, Biglieri et al. (32), Scaroni et al. (33), and Oshiro et al. (34) also reported that only female individuals were affected and that deficiency of *CYP17A1* also caused infertility. Therefore, we hypothesize that the measurement of blood flow using our system in zebrafish larvae, performed 3 days post-fertilization, may not represent the entirety of the effects of this gene, as sex determination and differentiation (which are vital to the role of *cyp17a1* deficiency in human hypertension) only take place at 21–23 days post-fertilization in zebrafish (35). Nevertheless, we can report that *cyp17a1*-deficient zebrafish (both heterozygotes and homozygotes) were able to reproduce normally.

Both *nt5c2* gene paralogs in zebrafish are reported to have similar phenotypes in the OMIM database. Most available literature regarding the human *NT5C2* gene suggests that it has a dominant role in spastic paraplegia 45 (SPG45) (36) and ALL (37). A common known phenotype associated with ALL is that it leads to the formation of immature or undeveloped blood cells (38). The blood smears staining images from *nt5c2a* mutant zebrafish showed increased number of immature or undeveloped blood cells compare to wild types. As explained above, the platform we employed to measure blood-flow parameters computes the number and speed of blood cell movements in a defined area; therefore, the increased number of immature blood cells floating in the vasculature of *nt5c2a* mutant zebrafish could arguably have influenced the high blood-flow parameters detected by the system. Hence, we propose that the high blood-flow parameters measured in the *nt5c2a* mutants may not demonstrate direct causality of this gene for the phenotype, but rather may be due to the ALL kind phenotype associated with *nt5c2* gene. On the other hand, increased expression of hypertension and inflammatory markers, i.e., *ace* and *crp* in the *nt5c2 “a” and “b”* mutants (Table 2) points toward its role in the increased blood-flow parameters. Here, we understand that the *nt5c2* gene warrants more investigations to unravel complete mechanistic pathway behind the increased blood-flow parameters in zebrafish mutants.

In the human family study, deficiency of *CNNM2* has been identified in patients suffering from intellectual disability, seizures, hypomagnesemia, infertility, and altered blood pressure (39). Arjona et al. reported the role of *cnnm2* gene isoforms in zebrafish using a morpholino knockdown model that caused disturbed brain development, increased embryonic spontaneous contractions, weak touch-evoked escape behavior, and reduced magnesium content, indicating an impairment of renal Mg^2+^ absorption (40). In another study, *CNNM2* systemic heterozygotes and kidney specific homozygotes mice were reported with hypomagnesemia leading to have significantly reduced blood pressure (41). Here, we report increased blood flow, linear velocity, and arterial pulse in morpholino knockdowns of both *cnnm2* isoforms in zebrafish. To further investigate the mechanism underlying the observed high blood-flow phenotype, we first validated our morpholino knockdown results in the *cnnm2a* zebrafish mutants. Subsequently, we detected impaired renal function in *cnnm2a* zebrafish mutants by renal assay. The establishment of impaired renal function in *cnnm2a* mutants leads to the obvious question of whether the functions of renin or aldosterone, which are the key regulators of hypertension via the RAAS pathway, are altered in this model. Since it is well-established that zebrafish do not synthesize aldosterone (42) (the main mammalian mineralocorticoid), we measured renin by ELISA assay and found that its levels were increased in lysates of *cnnm2a* zebrafish mutant larvae. Expression of the *ren* gene (encoding renin in zebrafish), along with the inflammation and hypertension markers (*ace* and *crp*), was found significantly higher than that in AB wild-type controls. Overall, we conclude that Mg^2+^ depletion (40), increased levels of renin, and increased expression of the *ren* gene trigger the RAAS pathway in *cnnm2a* zebrafish mutants, leading to increased blood-flow parameters or hypertension.

### Limitations of the Study

We understand the limitation of the current study, as well as to highlight them. (1) The 10q24.32 selected locus was identified from the GWAS in European population, therefore it might be a possibility that the mechanistic pathways suggested here may not completely be translatable to other ethnicities or ancestries. (2) *cyp17a1* zebrafish mutants need to be investigated via other methods where blood flow or pressure parameters can be determined from more developed larvae (after sex maturation). (3) A zebrafish transgenic reporter line can be used for kidney [*Tg(wt1b:EGFP)*] and blood cells (*sd2:Tg*) in future studies for understanding the underlying mechanisms of topics we touched briefly such as co-regulation of *cnnm2a* and *nt5c2a* genes, kidney function, and its involvement in *cnnm2a* mutants.

In summary, we dissected the 10q24.32 locus associated with high BP in GWAS. We uncovered different potential mechanisms leading to the high blood-flow phenotype in *nt5c2* and *cnnm2* mutant zebrafish. Furthermore, this study could serve as a blueprint for dissection and investigation of additional loci associated with BP, such as 13q34, 17q11.2, and 7p13, which is ongoing in our laboratory.

## Data Availability Statement

All datasets generated for this study are included in the article/Supplementary Material.

## Ethics Statement

Ethical approval of the animal study was waived in accordance with the recommendations of guidelines EU Directive 2010/63/EU set by European Commission, according to this legislation Embryos and larvae up to 5 days old are excepted. The legislation criterion is: independently feeding larval forms (Directive 2010/63/EU), in other words; when the larvae are able to move through the water column independently, when their digestive tract is functional, and when they are beginning to hunt for prey which has also been simplified and published in PMID: 21726626.

## Author Contributions

KV conceived, designed, performed, and collected the data and wrote the manuscript. CH, KT, and JR contributed in the critical analysis of the data and manuscript revisions. ZA and JE supervised the project, critically analyzed the data, reviewed the manuscript, and helped in designing the initial concept of the project. All authors contributed to the article and approved the submitted version.

## Conflict of Interest

The authors declare that the research was conducted in the absence of any commercial or financial relationships that could be construed as a potential conflict of interest.
