# Supplementary material for: Studies in Zebrafish Demonstrate That CNNM2 and NT5C2 Are Most Likely the Causal Genes at the Blood Pressure-Associated Locus on Human Chromosome 10q24.32
Source: Front Cardiovasc Med. 2020 Sep 2;7:135. doi: 10.3389/fcvm.2020.00135 (PMC7492806; doi:10.3389/fcvm.2020.00135)
Supplement: Supplementary file 1 [file Data_Sheet_1.docx]

**Studies in zebrafish demonstrate that *CNNM2* and *NT5C2* are most likely the causal genes at the blood pressure-associated locus on human chromosome 10q24.32**

K.K. Vishnolia^1,2,3^, C. Hoene^1,2,3^, K. Tarhbalouti^1,2,3^, J. Revenstorff^1,2,3^, Z. Aherrahrou^1,2,3^, J. Erdmann^1,2,3, *^

**Supplementary file 1**

PCR conditions mentioned below for genotyping and qPCR

**(Taqman) HT7900 qPCR program**

50°C 2min

95°C 10min

95°C 15sec |

60°C 1min | x40

95°C 15sec

60°C 15sec

95°C 15sec

**(Taqman) qPCR reaction**

3.75µl PowerUp qPCR Mastermix with SybrGreenI

1.125µl dd Water

1.125µl Primer F+R (5pmol/µl)

1.5µl cDNA (5-10 ng/µl)

**Genotyping PCRs**

**NT5C2A**

- Primer: zfNT5C2A-RFLP
- PCR: TD62s30: 94°C 3min

94°C 30s |

62°C 30s |

72°C 30s | x3

94°C 30s |

60°C 30s |

72°C 30s | x3

94°C 30s |

58°C 30s |

72°C 30s | x3

94°C 30s |

56°C 30s |

72°C 30s | x35

72°C 10min

- PCR: Bioline 5xBuffer 3.0µl

H2O 9.4µl

MyTaq 0.1µl

DNA 1.0µl

Primer F (10µM) 0.75µl

Primer R (10µM) 0.75µl

- RFLP : 10xFast Digest Buffer 2.5µl

20xSAM 1.25µl

Water 5.25µl

BseMII 1.0µl

PCR reaction 15µl

- incubation at 55°C for 2h, then agarose gel 2%
- G-Allel: 215, 111, 32, 205bp
- T-Allel: 215, 143, 205bp

**CNNM2A**

- Primer: zfCNNM2a-RFLP
- PCR: TD64s30: 94°C 3min

94°C 30s |

64°C 30s |

72°C 30s | x3

94°C 30s |

62°C 30s |

72°C 30s | x3

94°C 30s |

60°C 30s |

72°C 30s | x3

94°C 30s |

58°C 30s |

72°C 30s | x35

72°C 10min

- PCR: TaKaRa GCI buffer 5.0µl

dNTPs 0.8µl

H2O 2.3µl

MyTaq 0.1µl

DNA 1.0µl

Primer F (10µM) 0.5µl

Primer R (10µM) 0.5µl

- RFLP : 10xFast Digest Buffer 2.0 µl

Water 17.0µl

TaqI (10u/µl) 1.0 µl

PCR reaction 10.0µl

- incubation at 65°C for 3h, then agarose gel 2%
- KO: 553bp
- WT: 218, 335bp

**CYP17A1**

- Primer: zfCyp17A1-seq
- PCR: TD61s30: 94°C 3min

94°C 30s |

61°C 30s |

72°C 30s | x3

94°C 30s |

59°C 30s |

72°C 30s | x3

94°C 30s |

57°C 30s |

72°C 30s | x3

94°C 30s |

55°C 30s |

72°C 30s | x35

72°C 10min

- PCR: Bioline 5xBuffer 2.0µl

H2O 6.4µl

MyTaq 0.1µl

DNA 0.5µl

Primer F (10µM) 0.5µl

Primer R (10µM) 0.5µl

- then sequencing with F-Primers
