# Supplementary material for: Studies in Zebrafish Demonstrate That CNNM2 and NT5C2 Are Most Likely the Causal Genes at the Blood Pressure-Associated Locus on Human Chromosome 10q24.32
Source: Front Cardiovasc Med. 2020 Sep 2;7:135. doi: 10.3389/fcvm.2020.00135 (PMC7492806; doi:10.3389/fcvm.2020.00135)

**Studies in zebrafish demonstrate that *CNNM2* and *NT5C2* are most likely the causal genes at the blood pressure-associated locus on human chromosome 10q24.32**

K.K. Vishnolia^1,2,3^, C. Hoene^1,2,3^, K. Tarhbalouti^1,2,3^, J. Revenstorff^1,2,3^, Z. Aherrahrou^1,2,3^, J. Erdmann^1,2,3, *^

**Supplementary file 2**

Survival plot below represent results from Ramipril treatment of n= 25 zebrafish larvae in each group for different concentrations i.e. 0.1, 0.2, 0.3, 0.4 and 0.5 nM from day 0 till day 5.


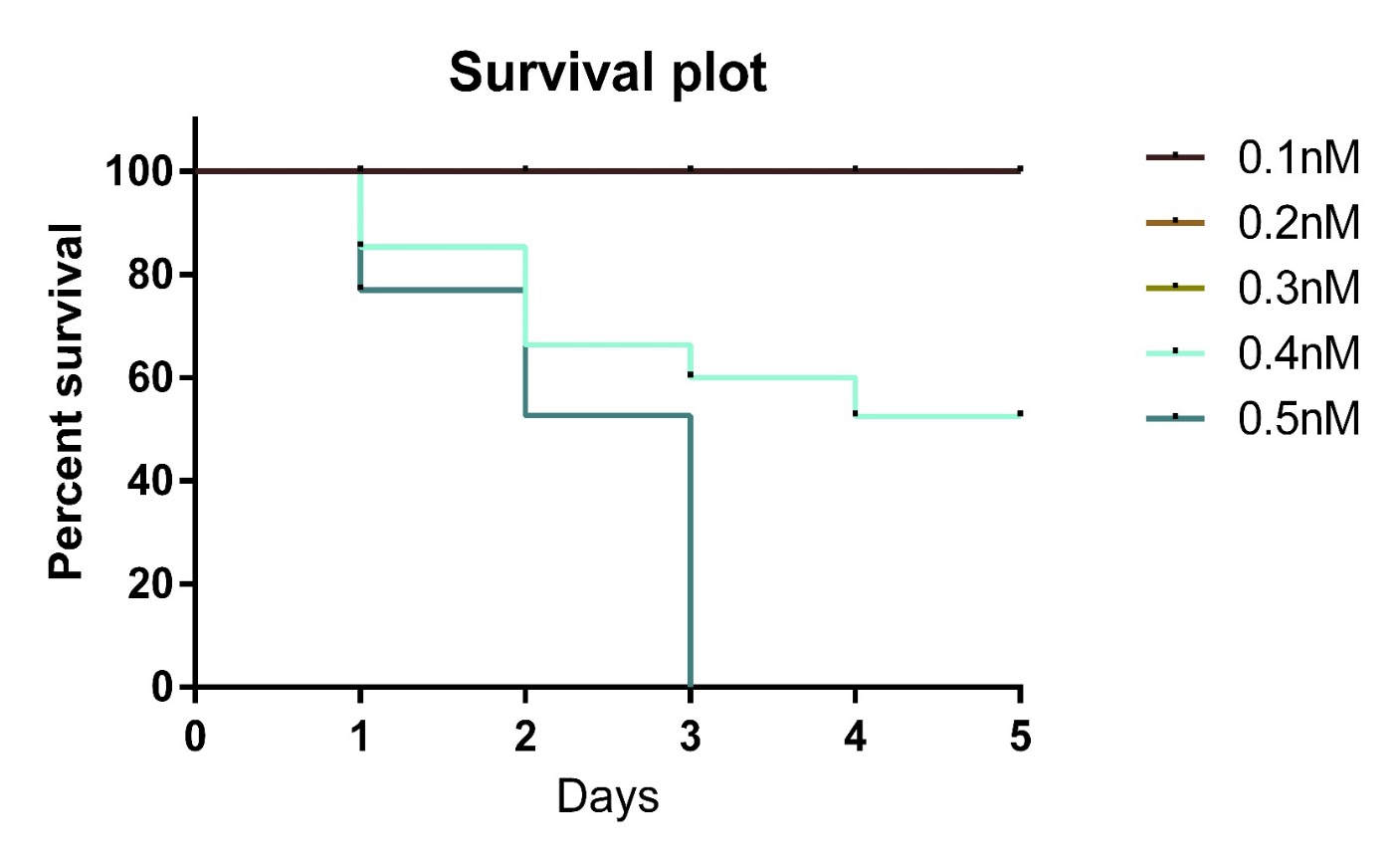

Supplement: Supplementary file 2 [file Data_Sheet_2.docx]
